# Fructose potentiates the protective efficiency of live *Edwardsiella tarda* cell vaccine

**DOI:** 10.3389/fimmu.2023.1170166

**Published:** 2023-03-30

**Authors:** Chao Wang, Xuan-xian Peng, Hui Li

**Affiliations:** ^1^ State Key Laboratory of Bio-Control, Southern Marine Science and Engineering Guangdong Laboratory (Zhuhai), Guangdong Key Laboratory of Pharmaceutical Functional Genes, School of Life Sciences, Sun Yat-sen University, University City, Guangzhou, China; ^2^ Laboratory of Freshwater Genetics and Breeding, Shandong Freshwater Fisheries Research Institute, Jinan, China; ^3^ Laboratory for Marine Fisheries Science and Food Production Processes, Qingdao National Laboratory for Marine Science and Technology, Qingdao, China

**Keywords:** *Edwardsiella tarda*, fructose, whole-cell vaccine, metabolomics, live vaccine

## Abstract

Vaccination is an effective measure to prevent infection by pathogens. Live vaccines have higher protective efficacy than inactivated vaccines. However, how live vaccines interact with the host from a metabolic perspective is unknown. The present study aimed to explore whether a live *Edwardsiella tarda* vaccine regulates host metabolism and whether this regulation is related to the protective efficacy of the vaccine. Therefore, a gas chromatography mass spectrometry (GC-MS)-based metabolomics approach was used to investigate the metabolomic profile of mice serum after vaccination with live *E. tarda* vaccine. Fructose was identified as a key biomarker that contributes to the immune protection induced by the live vaccine. Moreover, co-administration of exogenous fructose and the live vaccine synergistically promoted survival of mice and fish after bacterial challenge. These results indicate that metabolites, especially fructose, can potentiate the live *E. tarda* vaccine to increase its protective efficiency.

## 1 Introduction

*Edwardsiella tarda* is a Gram-negative bacterial species that causes infectious disease called edwardsiellosis in fish, reptiles, birds, and mammals (1, 2). Symptoms include gastroenteritis, peritonitis, meningitis, wound infections, and septicemia (3, 4). In the aquaculture industry, edwardsiellosis results in great economic losses all over the world (5, 6). Antibiotics are an effective approach to treat edwardsiellosis, but antibiotic overuse and misuse result in frequent isolation of antibiotic-resistant *E. tarda* (7, 8). These antibiotic-resistant *E. tarda* strains are insensitive to antibiotics. Therefore, alternative methods are needed to control these bacteria. Among them, vaccination is an effective way to prevent bacterial infection with few side effects (9–11). Therefore, a vaccine approach would be highly valuable.

Among the multiple types of vaccines, whole-cell vaccines were developed first but are still used (12, 13). Whole-cell vaccines include live and inactivated vaccines. As inactivated vaccines are unable to replicate *in vivo*, this type of vaccine is considered to be safer than live vaccines (14). However, live vaccines have higher protective efficacy than inactivated vaccines as live vaccines can be better recognized by the immune system (15, 16). Thus, improving the efficacy of a live *E. tarda* vaccine is a key issue for the development of a high-quality live *E. tarda* vaccine.

Metabolic modulation plays a role in immune response against bacterial pathogens (17–19). Several lines of evidence have shown that metabolites, such as linoleic acid, leucine, N-acetylglucosamine, glucose, malic acid, palmitic acid, and glycine, increase hosts’ survival of *Vibrio alginolyticus*, *Streptococcus iniae*, and *E. tarda* infection (20–27). The protective efficacy of live vaccines against *E. tarda* infection has been reported to be related to increased biosynthesis of palmitic acid (27). These results motivated us to explore whether metabolites potentiate the protective efficacy of a live *E. tarda* vaccine.

## 2 Materials and methods

### 2.1 Ethics statement

All work was conducted in strict accordance with the recommendations of the Guide for the Care and Use of Laboratory Animals of the US National Institutes of Health. The protocol was approved by the Institutional Animal Care and Use Committee of Sun Yat-sen University (animal welfare assurance number: 16).

### 2.2 Bacterial strains and animals

Bacterial strain *E. tarda* EIB202 was obtained from Prof. Yuanxin Zhang at East China University of Science and Technology. The strain was grown in tryptic soy broth (TSB) medium at 30°C and harvested when the optical density at 600 nm (OD_600_) reached 1.0. SPF Kunming mice (20.2 ± 1.32g) were provided by the Animal Center of Sun Yat-sen University and fed twice a day with a dry pellet diet along with sterile water. *Micropterus salmoides* (27.8 ± 2.36g) were purchased from a commercial breeding corporation (Guangzhou Mingfeng Fisheries Co., Ltd, Guangzhou, P.R. China) and maintained in 25-L open-circuit water tanks with aeration. The animals were fed with 3% of their body weight/day. After acclimating for 1 week, the animals were randomly divided into several groups to investigate the effect of the live vaccine.

### 2.3 Preparation of live vaccine

Vaccines were prepared as previously described (13). In brief, a single colony of *E. tarda* EIB202 was picked from a TSB plate and cultured overnight in TSB medium at 30°C. The cultures were diluted 1:100 in fresh TSB medium and grown at 30°C. Bacterial cells with an OD_600_ of 1.0 were harvested by centrifugation at 4,000 g for 15 min and washed three times with saline. The cells were resuspended in sterile saline, and the solution was used as the live *E. tarda* vaccine.

### 2.4 Protective efficacy of live vaccine

Twenty mice were divided into two groups, live vaccine and PBS control. The live vaccine was intraperitoneally injected into mice at 10^6^ CFU/mouse (live vaccine group), with PBS being used as the control (PBS control). After two injections at an interval of 7 days, these mice were challenged by 5x10^8^ CFU of EIB202 and observed twice daily for 7 days.

### 2.5 Administration of vaccine and collection of plasma samples

The live vaccine was administered to mice at 10^6^ CFU/mouse by intraperitoneal injection, with PBS being used as the control. After two injections at an interval of 7 days, blood was drawn from the orbit of the live mice, and sodium citrate was added as an anticoagulant. Plasma was collected by centrifugation at 3,000 g and 4°C for 10 min. Metabolites were extracted from 50 μL plasma with 0.2 mL cold methanol (Sigma, USA) containing 10 μL 0.1 mg/mL ribitol (Sigma, USA) as an analytical internal standard for normalization across samples. After centrifugation at 12,000 g and 4°C for 10 min, 0.1 mL supernatant was collected and dried using a vacuum centrifugation device (LABCONCO, USA). The dried samples were used for gas chromatography mass spectrometry (GC-MS) analysis, involving six biological samples with two technical repeats per group.

### 2.6 GC-MS analysis

Samples were processed by derivatization involving a two-stage technique, as previously described (28). In brief, 20 μL of 40 mg/L methoxyamine hydrochloride in pyridine (Sigma) was added to the dried samples for 90 min at 37°C. Thereafter, 80 μL N-methyl-N-(trimethylsily)trifluoroacetamide (MSTFA, Sigma, USA) with 1% trimethylchlorosilane (TMCS) was mixed and reacted with the samples for 30 min at 37°C.

GC-MS analysis was carried out using an Agilent 7890A gas chromatograph equipped with an Agilent 5975C VL MSD detector (Agilent Technologies, USA). 1 μL sample was injected into a DB-5MS column (30 m length × 250 μm i.d. × 0.25 μm thickness) with splitless injection and the flow rate of carrier gas (helium) was 1 mL/min. The initial temperature of the gas chromatograph oven was 85°C for 5 min followed by an increase to 270°C at a rate of 15°C/min, and then the temperature was held at 270°C for 5 min. The mass spectrometer was operated in the range of 50–600 m/z.

### 2.7 Data processing and statistical analysis

Mass spectra were analyzed based on the Total ion chromatogram (TIC) by XCalibur 2.1 software (Thermo Fisher Scientific), and compounds were identified using the National Institute of Standards and Technology (NIST) library and NIST MS Search Program 2.0. Peaks from different samples were aligned based on retention time and the mass spectrum. After normalization to ribitol, the data on the peak areas were used for subsequent analysis. The normalized abundances of differential metabolites were used in a Z-score analysis, which was based on the sample value minus the mean and divided by the standard deviation. Principal component analysis (PCA) and orthogonal partial least squares discriminant analysis (OPLS-DA) were carried out using SIMCA 12.0 (Umetrics, Umeå, Sweden). Differential metabolites were used for pathway enrichment analysis using MetaboAnalyst 2.0 (29). Interactive Pathways (iPath) analysis was carried out by iPath3.0 (https://pathways.embl.de/).

### 2.8 Protective efficacy of live vaccine potentiated by fructose

Exogenous fructose was administered as previously described (25). In brief, Kuming mice and *Micropterus salmoides* were acclimatized at 28°C for 7 days. They were randomly divided into five groups of 30 or 60 per group, respectively. For both the mouse and fish groups, the animals in the five groups were injected twice at an interval of 7 days with 0.1 mL one of the following: (1) live vaccine, (2) live vaccine and 0.18 mg fructose, (3) live vaccine and 1.80 mg fructose, (4) 0.85% sterilized PBS, or (5) 1.80 mg fructose. The live vaccine was used at dosages of 10^6^ CFU/mouse or 4×10^3^ CFU/fish. Thereafter, the mice and fish were challenged by intraperitoneal inoculation of EIB202 (5×10^8^ CFU/mouse and 5×10^5^ CFU/fish) and observed twice daily for 15 and 7 days, respectively.

## 3 Results

### 3.1 Plasma metabolomic profiling of mice immunized with live *E. tarda* vaccine

**Figure 1:**
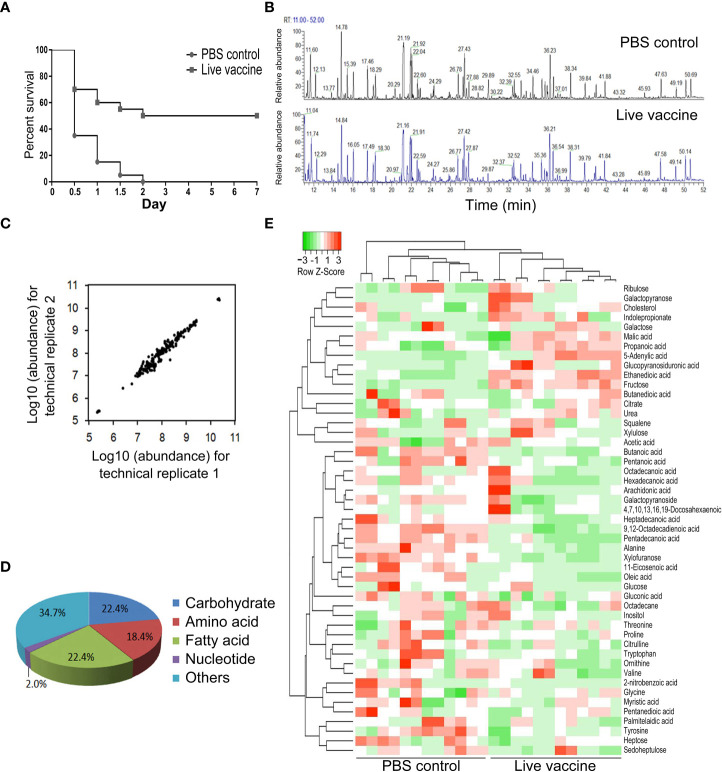
Metabolomic profiling of identified metabolites. **(A)** Survival of mice immunized by live *E*. *tarda* vaccine and then challenged by EIB202. **(B)** Representative total ion current chromatogram from control and live groups. **(C)** Reproducibility of metabolomic profiling platform. The correlation coeffcient is shown by two technical replicates of metabolite abundances. **(D)** Category of the identified metabolites. **(E)** Cluster analysis of metabolites. Heat map shows differential metabolites. Green and red color indicates decrease and increase of metabolites, respectively (see color scale).

Mice were immunized twice with live *E. tarda* vaccine and then challenged using *E. tarda* EIB202, which led to 50% survival (**Figure 1A**). Plasma was collected from the live mice to explore the association of metabolites with the protective efficacy of vaccine. A GC-MS-based metabolomics analysis was performed to profile the metabolic signature in the plasma after vaccination, with PBS being used as a control. A total of 130 aligned individual peaks were obtained from each sample (**Figure 1B**). After removing the artificial peaks, 49 metabolites were identified. Scatter plots of the metabolite abundances in pairs of technical repeats indicated correlation coefficients ranging between 0.993 and 0.999, demonstrating the reliability of the methods (**Figure 1C**). The metabolites were classified into five metabolic categories: carbohydrates (22.4%), amino acids (18.4%), fatty acids (22.4%), nucleotides (2.0%), and others (34.7%) (**Figure 1D**). Hierarchical clustering of the 49 metabolites is shown in (**Figure 1E**). These results indicate that vaccination induced a metabolic shift.

### 3.2 Differential abundance of metabolites

**Figure 2:**
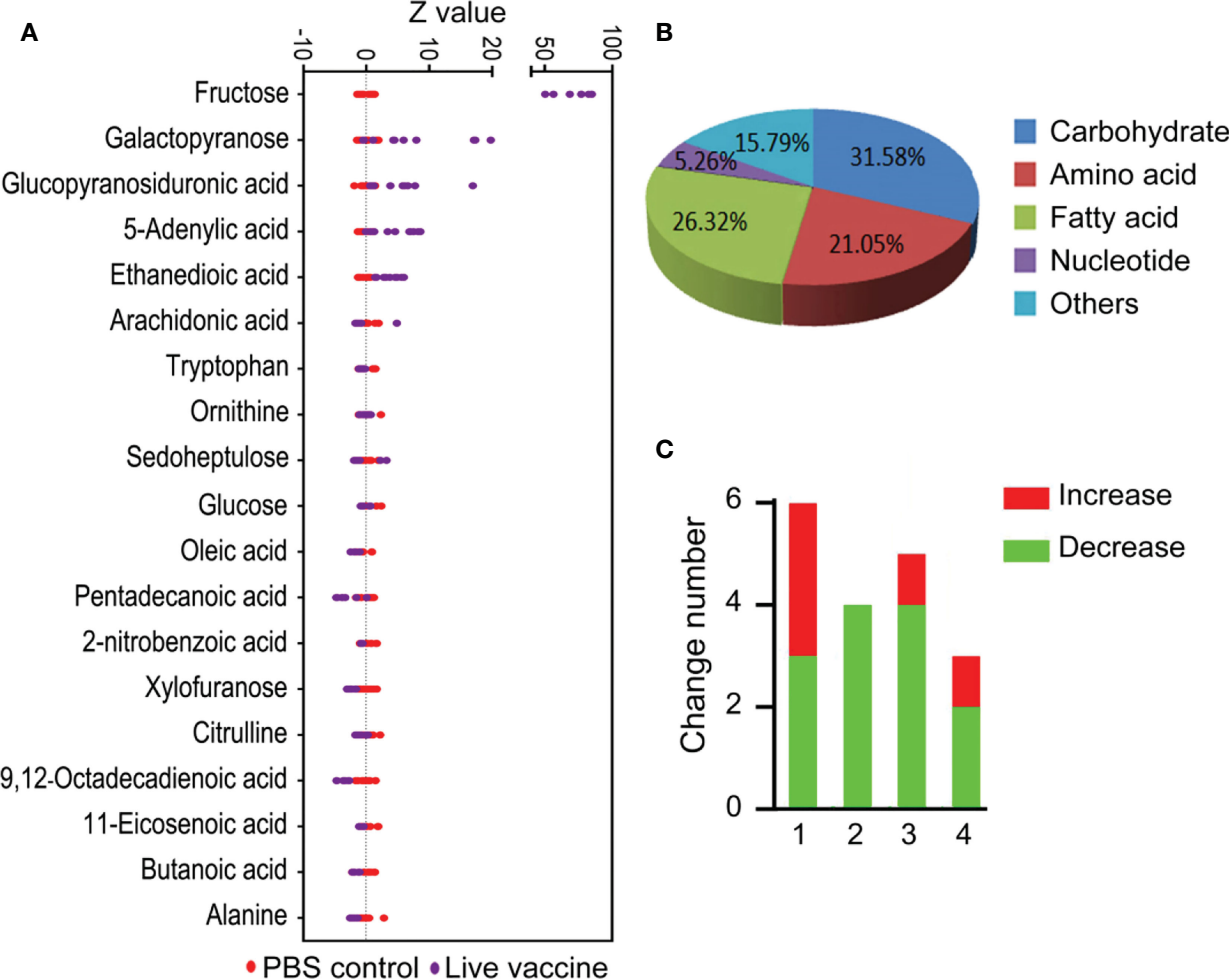
Differential metabolites analysis in response to vaccine stimulation. **(A)** Z-score plot of differential metabolites immunized with vaccine compared with PBS control. Each point represents one metabolite in one technical repeat and colored by sample types. **(B)** Category of different metabolites in live bacteria group. **(C)** Number of differential metabolites in live bacteria group. 1, carbohydrate; 2, amino acid; 3, fatty acid; 4, others.

There were 19 differential abundance of metabolites between the live vaccine group and PBS control group (**Figure 2A**). The Z-score plot spanned from −4.72 to 85.0 in the live vaccine group (**Figure 2A**), with fructose being the metabolite with the most increased abundance. Among the metabolites with differential abundances, 31.58% were carbohydrates, 21.05% were amino acids, 26.32% were fatty acids, 5.26% were nucleotides, and 15.79% were metabolites with unknown function (**Figure 2B**). There were more decreased amino acids and fatty acids in the live vaccine group than the PBS group, while the number of altered carbohydrates was similar between the two groups (**Figure 2C**). These results indicate that the live vaccine induced a differential metabolome, with fructose being the most elevated metabolite.

### 3.3 Metabolic biomarkers identified using multivariate analysis

**Figure 3:**
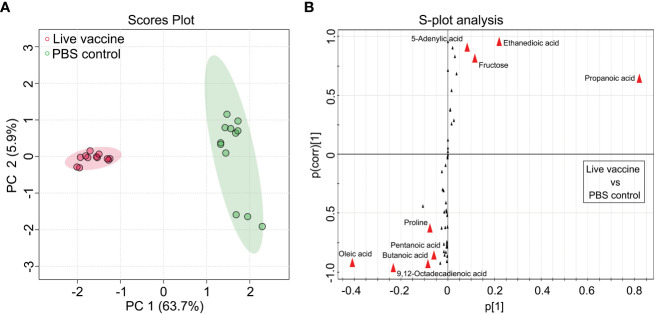
Identification of crucial metabolites. **(A)** The PCA analysis of the PBS control group and live vaccine group. Each dot represents the technique replicates in the plot. **(B)** S-plot generated from OPLS-DA. Circle represents individual metabolite, where potential biomarkers are highlighted with red.

OPLS-DA was used to identify potential metabolic biomarkers associated with the protective efficacy of the vaccine. In the PCA, PC1 separated the two groups (**Figure 3A**). An S-plot was used to identify discriminatory variables, and the following biomarkers were selected from component p[1]: increased fructose and ethanedioic acid, and decreased oleic acid, 5-adenylic acid, 9,12-octadecadienoic acid, butanoic acid, and alanine (**Figure 3B**). These results confirm that fructose is a key biomarker.

### 3.4 Enrichment of metabolic pathways

**Figure 4:**
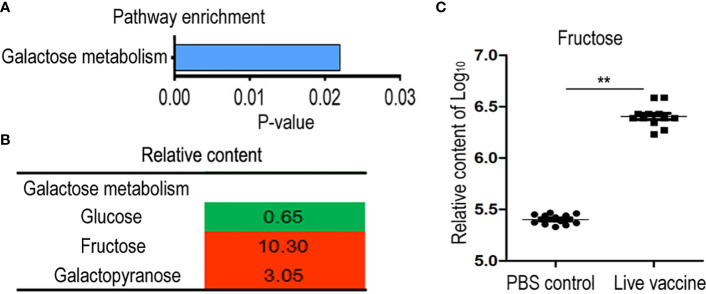
Metabolomics analysis of enriched pathway. **(A)** Pathway enrichment of different metabolites in live group. **(B)** Integrative analysis of metabolites in enriched pathways. The value was relative content in the live groups compared with control group. Red color and green color indicate increased and decreased metabolites, respectively. **(C)** The scatter diagram of different metabolite. The Y-axis is relative content of metabolites. **, P <0.01 using the Chi-square test.

Differential abundances of metabolites indicated that metabolic pathways were affected. Identifying the enriched pathways is helpful to identify the altered metabolic pathways. Galactose metabolism was enriched in the live vaccine group compared to the PBS group (**Figure 4A**). In this pathway, the metabolites with differential abundances included glucose, fructose, and galactopyranose (**Figure 4B**). Importantly, fructose was the metabolite with the most elevated abundance in the metabolic metabolism (**Figures 4B, C**). These results indicate that the live vaccine altered galactose metabolism.

**Figure 5:**
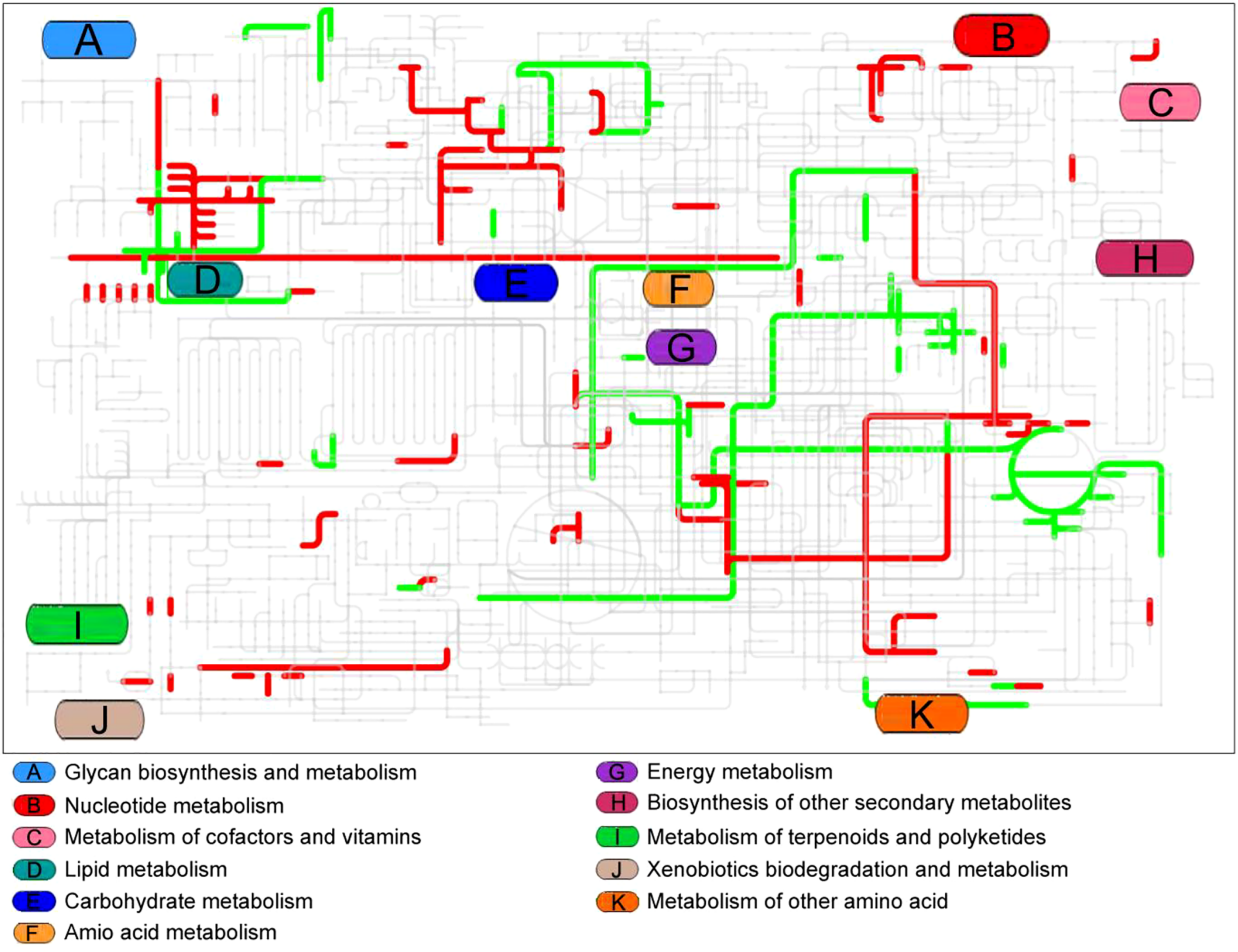
Integrated metabolomics analysis of pathway by iPath. The changed pathway in live group, compared with control group, was shown in color. Red lines represent increase and green lines indicate decrease.

Interactive Pathways Explorer (iPath) was used to provide an overview of the enriched metabolic pathways (**Figure 5**). Seven pathways were up-regulated in the live vaccine group compared to the PBS group: fructose and mannose metabolism, starch and sucrose metabolism, glycerolipid metabolism, arachidonic acid metabolism, purine metabolism, TCA cycle, and riboflavin metabolism. Two pathways were down-regulated: arginine and proline metabolism and alanine, aspartate, and glutamate metabolism. These results together suggest that the live vaccine altered metabolism to a large degree.

Notably, enrichment of metabolic pathways and iPath are two different analysis methods, which seems no overlapped metabolic pathways between the two identification. However, many metabolites in fructose and mannose metabolism, starch and sucrose metabolism, glycerolipid metabolism, glycerolipid metabolism are included in galactose metabolism (**Table 1**)(https://www.kegg.jp/kegg/kegg2.html).

**Table 1:** Metabolites related to galactose metabolism.

Fructose and mannose metabolism	Starch and sucrose metabolism	Glycerolipid metabolism
D-Fructose	UDP-glucose	UDP-glucose
Glycerone phosphate	D-Glucose	D-Glucose 1-phosphate
D-Glyceraldehyde 3-phosphate	D-Fructose 6-phosphate	Glycerone phosphate
D-Mannose	Sucrose	Glycerol
alpha-D-Glucose	D-Fructose	D-Glyceraldehyde
D-Glyceraldehyde	D-Glucose 1-phosphate	3-beta-D-Galactosyl-sn-glycerol
D-Sorbitol		
L-Rhamnulose 1-phosphate		

### 3.5 Fructose improves the protective efficiency of the live vaccine

The above results show that fructose was the most altered metabolite. Therefore, increased fructose may contribute to the protective efficacy of the live vaccine. A mouse model was used to confirm the effect of fructose on the vaccine protective efficacy. To do this, mice were injected with live vaccine (group 1), live vaccine and 0.18 or 1.80 mg fructose (groups 2 and 3, respectively), PBS (group 4), or 1.80 mg fructose (group 5). After two injections at an interval of 7 days, the mice were challenged with EIB202 and their survival was monitored for 15 days. All mice in groups 4 and 5 died within 2 days, whereas 50.0%, 56.7% and 76.7% survived in groups 1, 2, and 3, respectively (**Table 2**). There were significant differences between groups 3 and 4 (RPS1) and groups 3 and 1 (RPS2), suggesting the role of fructose. No significance was found between groups 2 and 3.

**Table 2:** Immune protection of live vaccine with fructose in mice.

group	Immunogen	Total mice	Survive No	ADR(%)	RPS1 (%)	RPS2 (%)
1	Live vaccine	30	15	50.0%	50.0%	
2	Live vaccine with 0.18mg Fructose	30	17	43.3%	56.7%	13.4%
3	Live vaccine with 1.8mg Fructose	30	23	23.3%	76.7%^*^	53.4% ^*^
4	PBS	20	0	100.0%		
5	1.8mg Fructose	20	0	100.0%		

All the treatment was same volume (0.1mL). Survived, the number of mice survived in the experiment. ADR, accumulating death rates; RPS, relative percent survival. RPS was calculated as RPS1 = 1 - (% mortality of Fructose and/or bacteria treated group/% mortality of control group) × 100. RPS2 = 1 - (% mortality of Fructose and bacteria treated group/% mortality of bacteria treated group) × 100. The experiments are repeated twice. ^*^ P < 0.05.

Furthermore, a *Micropterus salmoides* model was used to test the effect of fructose on the vaccine protective efficacy, using the same groupings as for the mice. The survival of the fish was monitored for 7 days. All fish in groups 4 and 5 died within 4 days, whereas 55.0%, 61.7%, and 76.7% survived in groups 1, 2, and 3, respectively (**Table 3**). There were significant differences in survival between groups 3 and 4 (RPS1) and groups 3 and 1 (RPS2), confirming the role of fructose. No significance was found between groups 2 and 3.

**Table 3:** Immune protection of live vaccine with fructose in *M. salmoides*.

Group	Immunogen	Total fish	Survive No	ADR(%)	RPS1 (%)	RPS2 (%)
1	Live vaccine	60	33	45.0%	55.0%	
2	Live vaccine with 0.18mg Fructose	60	37	38.3%	61.7%	14.8%
3	Live bacteria with 1.8mg Fructose	60	46	23.3%	76.7% ^*^	48.2%^*^
4	PBS	60	0	100.0%		
5	1.8mg Fructose	60	0	100.0%		

All the treatment was same volume (0.1mL). Survived, the number of ﬁsh survived in the experiment. ADR, accumulating death rates; RPS, relative percent survival. RPS was calculated as RPS1 = 1 - (% mortality of Fructose and/or bacteria treated group/% mortality of control group) × 100. RPS2 = 1 - (% mortality of Fructose and bacteria treated group/% mortality of bacteria treated group) × 100. The experiments are repeated twice. ^*^ P < 0.05.

## 4 Discussion

The present study explores the role of metabolites in vaccination. Comparing metabolome between the live vaccine group and PBS control group, the abundances of metabolites were significantly changed. Thus, the live vaccine induces a metabolic response, suggesting that the metabolism plays a role in the vaccine-induced immune protection. Pattern recognition analysis identified fructose as the most important biomarker. Furthermore, exogenous fructose increased the live vaccine’s protective efficiency against bacterial challenge in mouse and fish models. These results indicate that metabolites can promote the protective efficiency of live vaccine against bacterial challenge, and fructose was identified as an effective metabolite that potentiates live *E. tarda* vaccine. To our knowledge, this is the first report on a metabolite promoting a vaccine’s efficiency.

It has been found that a metabolomics approach is effective at identifying biomarkers. To explore which biomarkers are associated with an increase in the protective efficacy of the live *E. tarda* vaccine, a GC-MS-based metabolomics analysis was performed in the present study. The live *E. tarda* vaccine induced a differential metabolome, indicating that there is a live vaccine-induced metabolome. This is consistent with the recent findings that, according to their sensitivity to antibiotics, bacteria display either an antibiotic-resistance or antibiotic-sensitive metabolome (28, 30), while hosts exhibit either an anti-infective or infective metabolome based on their ability to combat the infection (21, 31–35). Based on the live vaccine-induced metabolome, fructose was identified as a key biomarker that contributes to the immune protection efficiency of the live vaccine. Indeed, exogenous fructose increased the live vaccine-mediated immune protection against bacterial challenge in mice and fish. Thus, positive feedback was found between the live vaccine and fructose. Specifically, the live vaccine increased fructose and the increased fructose potentiated the ability of the vaccine to stimulate immune protection. Recently, we showed that live and inactivated *E. tarda* vaccines stimulate the generation of differential abundances of palmitate that contribute to differential innate immunities against bacterial infection (27). These results together indicate that vaccine-induced immune protection is related to the vaccine’s alteration of the metabolome. Therefore, the findings highlight that metabolites could be used as vaccine adjuvants.

Fructose is an important metabolite used for energy supply (36) and is involved in host–pathogen interactions (37, 38). Exogenous fructose decreases bacterial growth and biofilm formation (39). Antibiotic-resistant bacteria have a lower level of fructose than antibiotic-sensitive bacteria, and exogenous fructose has been shown to promote kanamycin-mediated killing (40). Fructose modulates the host’s immune response by increasing the expression of monocyte chemoattractant protein 1 and uric acid (37, 38). Monocyte chemoattractant protein 1 is a chemoattractant for inflammatory monocytes and dendritic cells, and uric acid activates the inflammasome pathway to release interleukin (IL)-1β, which enhances inflammation (41, 42). Furthermore, fructose activates dendritic cells *via* IL-6 and induces interferon-γ secretion by T cells (43). Therefore, fructose may activate the immune response as an immunologic adjuvant, indicating that fructose could be used as an adjuvant to increase the protection efficiency of vaccines.

## 5 Conclusion

In summary, this study provides insights into a new strategy to increase the protective efficacy of a live vaccine. This strategy involved using a metabolomics analysis to identify biomarkers that contribute to the protective efficiency of a vaccine and then administering a key biomarker with the vaccine in order to enhance the vaccine’s protective effects. Fructose was identified as the most crucial biomarker and found to be a metabolite that promotes protection against *E. tarda* infection. These findings highlight a way to identify vaccine adjuvants and how to improve whole-cell vaccines.

## Data availability statement

The original contributions presented in the study are included in the article/supplementary material. Further inquiries can be directed to the corresponding author.

## Ethics statement

The animal study was reviewed and approved by the Institutional Animal Care and Use Committee of Sun Yat-sen University (animal welfare assurance number: 16).

## Author contributions

HL conceptualized and designed the project. HL and X-XP wrote the manuscript. CW performed experiments. All authors contributed to the article and approved the submitted version.

## References

[1] Song M Xie J Peng XX Li H. Identifcation of protective immunogens from extracellular secretome of *Edwardsiella tarda* . Fish Shellfsh Immunol (2013) 35:1932–6. doi: 10.1016/j.fsi.2013.09.033 24099803

[2] Sun L Chen H Lin W Lin X. Quantitative proteomic analysis of *Edwardsiella tarda* in response to oxytetracycline stress in biofilm. J Proteomics (2017) 150:141–8. doi: 10.1016/j.jprot.2016.09.006 27638425

[3] Crosby SN Snoddy MC Atkinson CT Lee DH Weikert DR. Upper extremity myonecrosis caused by *Edwardsiella tarda* resulting in transhumeral amputation: case report. J Handb Surg-Am (2013) 38:129–32. doi: 10.1016/j.jhsa.2012.10.009 23200948

[4] Suezawa C Yasuda M Negayama K Kameyama T Hirauchi M Nakai T. Identifcation of genes associated with the penetration activity of the human type of *Edwardsiella tarda* EdwGII through human colon epithelial cell monolayers. Microb Pathog (2016) 95:148–56. doi: 10.1016/j.micpath.2016.04.007 27057670

[5] Xu TT Zhang XH. *Edwardsiella tarda*: an intriguing problem in aquaculture. Aquaculture (2014) 431:129–35. doi: 10.1016/j.aquaculture.2013.12.001

[6] Loch TP Hawke J Reichley S Faisal M Piero FD Grifn MJ. Outbreaks of edwardsiellosis caused by *Edwardsiella piscicida* and *Edwardsiella tarda* in farmed barramundi (*Lates calcarifer*). Aquaculture (2017) 481:202–10. doi: 10.1016/j.aquaculture.2017.09.005

[7] Yu JE Cho MY Kim JW Kang HY. Large Antibiotic-resistance plasmid of *Edwardsiella tarda* contributes to virulence in fish. Microb Pathog (2012) 52(5):259–66. doi: 10.1016/j.micpath.2012.01.006 22342431

[8] Stock I Wiedemann B. Natural antibiotic usceptibilities of *Edwardsiella tarda*, e. ictaluri, and *E. hoshinae* . Antimicrob Agents Chemother (2001) 45(8):2245–55. doi: 10.1128/AAC.45.8.2245-2255.2001 PMC90638 11451681

[9] Czinn SJ Blanchard T. Vaccinating against *Helicobacter pylori* infection. Nat Rev Gastro Hepat (2011) 8:133–40. doi: 10.1038/nrgastro.2011.1 21304478

[10] Aagaard C Hoang T Dietrich J Cardona PJ Izzo A Dolganov G. A multistage tuberculosis vaccine that confers efficient protection before and after exposure. Nat Med (2011) 17:189–94. doi: 10.1038/nm.2285 21258338

[11] Nguyen HT Thu Nguyen TT Tsai MA Ya-Zhen E Wang PC Chen SC. A formalin-inactivated vaccine provides good protection against vibrio harveyi infection in orange-spotted grouper (*Epinephelus coioides*). Fish Shellfish Immunol (2017) 65:118–26. doi: 10.1016/j.fsi.2017.04.008 28419854

[12] Svennerholm AM Lundgren A. Recent progress toward an enterotoxigenic *Escherichia coli* vaccine. Expert Rev Vaccines (2012) 11:495–507. doi: 10.1586/erv.12.12 22551034

[13] Wang C Peng B Li H Peng XX. TolC plays a crucial role in immune protection conferred by *Edwardsiella tarda* whole-cell vaccines. Sci Rep (2016) 6:29488. doi: 10.1038/srep29488 27406266 PMC4942608

[14] Liu X Yang MJ Wang SN Xu D Li H Peng XX. Differential antibody responses to outer membrane proteins contribute to differential immune protections between live and inactivated *Vibrio parahemolyticus* . J Proteome Res (2018) 17(9):2987–94. doi: 10.1021/acs.jproteome.8b00176 30095909

[15] Yamasaki M Araki K Maruyoshi K Matsumoto M Nakayasu C Moritomo T. Comparative analysis of adaptive immune response after vaccine trials using live attenuated and formalin-killed cells of *Edwardsiella tarda* in ginbuna crucian carp (*Carassius auratus langsdorfi*). Fish Shellfsh Immunol (2015) 45:437–42. doi: 10.1016/j.fsi.2015.04.038 25959574

[16] Jeffrey BU Ulrich V Rino R. Vaccine manufacturing: challenges and solutions. Nat Biotechnol (2006) 24:1377–83. doi: 10.1038/nbt1261 17093488

[17] Young T Alfaro AC. Metabolomic strategies for aquaculture research: a primer. Rev Aquacult (2016) 10:26–56. doi: 10.1111/raq.12146

[18] Boothby M Rickert RC. Metabolic regulation of the immune humoral response. Immunity (2017) 46:743–55. doi: 10.1016/j.immuni.2017.04.009 PMC5640164 28514675

[19] Chen XH Liu SR Peng B Li D Cheng ZX Zhu JX. Exogenous l-valine promotes phagocytosis to kill multidrug-resistant bacterial pathogens. Front Immunol (2017) 8:207. doi: 10.3389/fimmu.2017.00207 28321214 PMC5337526

[20] Cheng ZX Ma YM Li H Peng XX. N-acetylglucosamine enhances survival ability of tilapias infected by *Streptococcus iniae* . Fish Shellﬁsh Immunol (2014) 40:524–30. doi: 10.1016/j.fsi.2014.08.008 25120218

[21] Peng B Ma YM Zhang JY Li H. Metabolome strategy against *Edwardsiella tarda* infection through glucose enhanced metabolic modulation in tilapias. Fish Shellﬁsh Immunol (2014) 45:869–76. doi: 10.1016/j.fsi.2015.06.004 26057462

[22] Zhao XL Wu CW Peng XX Li H. Interferon-2b against microbes through promoting biosynthesis of unsaturated fatty acids. J Proteome Res (2014) 13:4155–63. doi: 10.1021/pr500592x 25058871

[23] Ma YM Yang MJ Wang SY Li H Peng XX. Liver functional metabolomics discloses an action of l-leucine against *Streptococcus iniae* infection in tilapias. Fish Shellﬁsh Immunol (2015) 45:414–21. doi: 10.1016/j.fsi.2015.04.037 25957884

[24] Zeng ZH Du CC Liu SR Li H Peng XX Peng B. Glucose enhances tilapia against *Edwardsiella tarda* infection through metabolome reprogramming. Fish Shellfish Immunol (2017) 61:34–43. doi: 10.1016/j.fsi.2016.12.010 27965164

[25] Yang MJ Cheng ZX Jiang M Zeng ZH Peng B Peng XX. Boosted TCA cycle enhances survival of zebrafish to *Vibrio alginolyticus* infection. Virulence (2018) 9(1):634–44. doi: 10.1080/21505594.2017.1423188 PMC5955478 29338666

[26] Cheng ZX Guo C Chen ZG Yang TC Zhang JY Wang J. Glycine, serine and threonine metabolism confounds efficacy of complement-mediated killing. Nat Commun (2019) 10:3325. doi: 10.1038/s41467-019-11129-5 31346171 PMC6658569

[27] Xu D Wang J Guo C Peng XX Li H. Elevated biosynthesis of palmitic acid is required for zebrafish against *Edwardsiella tarda* infection. Fish Shellfish Immunol (2019) 92:508–18. doi: 10.1016/j.fsi.2019.06.041 31247319

[28] Peng B Su YB Li H Han Y Guo C Tian YM. Exogenous alanine and/or glucose plus kanamycin kills antibiotic-resistant bacteria. Cell Metab (2015) . 21(2):249–62. doi: 10.1016/j.cmet.2015.01.008 25651179

[29] Xia J Mandal R Sinelnikov IV Broadhurst D Wishart DS. MetaboAnalyst 2.0-a comprehensive server for metabolomic data analysis. Nucleic Acids Res (2012) 40:127–33. doi: 10.1093/nar/gks374 PMC3394314 22553367

[30] Jiang M Su YB Ye JZ Li H Kuang SF Wu JH. Ampicillin-controlled glucose metabolism manipulates the transition from tolerance to resistance in bacteria. Sci Adv (2023) 9:eade8582. doi: 10.1126/sciadv.ade8582 36888710 PMC9995076

[31] Zhao XL Chen ZG Yang TC Jiang M Wang J Cheng ZX. Glutamine promotes antibiotic uptake to kill multidrug-resistant uropathogenic bacteria. Sci Transl Med (2021) 13:eabj0716. doi: 10.1126/scitranslmed.abj0716 34936385

[32] Zhang S Wang J Jiang M Peng XX Li H. Reduced redox-dependent mechanism and glucose-mediated reversal in gentamicin-resistant *Vibrio alginolyticus* . Environ Microbiol (2019) 21(12):4724–39. doi: 10.1111/1462-2920.14811 31595636

[33] Kou TS Wu JH Chen XW Chen ZG Zheng J Peng B. Exogenous glycine promotes oxidation of glutathione and restores sensitivity of bacterial pathogens to serum-induced cell death. Redox Biol (2022) 58:102512. doi: 10.1016/j.redox.2022.102512 36306677 PMC9615314

[34] Jiang M Yang LF Chen ZG Lai SS Zheng J Peng B. Exogenous maltose enhances zebrafish immunity to levofloxacin-resistant *Vibrio alginolyticus* . Microb Biotechnol (2020) 13:1213–27. doi: 10.1111/1751-7915.13582 PMC7264874 32364684

[35] Jiang M Chen ZG Li H Zhang TT Yang MJ Peng XX. Succinate and inosine coordinate innate immune response to bacterial infection. PloS Pathog (2022) 18(8):e1010796. doi: 10.1371/journal.ppat.1010796 36026499 PMC9455851

[36] Havel PJ. Dietary fructose: implications for dysregulation of energy homeostasis and lipid/carbohydrate metabolism. Nutr Rev (2005) 63:133–57. doi: 10.1301/nr.2005 15971409

[37] Nakagawa T Hu H Zharikov S Tuttle KR Short RA Glushakova O. A causal role for uric acid in fructose-induced metabolic syndrome. Am J PhysiolRenal Physiol (2006) 290:625–31. doi: 10.1152/ajprenal.00140.2005 16234313

[38] Cirillo P Gersch MS Mu W Scherer PM Kim KM Gesualdo L. Ketohexokinase-dependent metabolism of fructose induces proinfammatory mediators in proximal tubular cells. J Am Soc Nephrol (2009) 20:545–53. doi: 10.1681/ASN.2008060576 PMC2653686 19158351

[39] Durmus NG Taylor EN Inci F Kummer KM Tarquinio KM Webster TJ. Fructose-enhanced reduction of bacterial growth on nanorough surfaces. Int J Nanomed (2012) 7:537–45. doi: 10.2147/IJN.S27957 PMC3273985 22334783

[40] Su YB Peng B Han Y Li H Peng XX. Fructose restores susceptibility of multidrug-resistant edwardsiella tarda to kanamycin. J Proteome Res (2015) 14(3):1612–20. doi: 10.1021/pr501285f 25675328

[41] Rock KL Kataoka H Lai JJ. Uric acid as a danger signal in gout and its comorbidities. Nat Rev Rheumatol (2013) 9:13–23. doi: 10.1038/nrrheum.2012.143 22945591 PMC3648987

[42] Petrasek J Iracheta-Vellve A Saha B Satishchandran A Kodys K Fitzgerald KA. Metabolic danger signals, uric acid and ATP, mediate infammatory cross-talk between hepatocytes and immune cells in alcoholic liver disease. J Leukoc Biol (2015) 98:249–56. doi: 10.1189/jlb.3AB1214-590R PMC4501673 25934928

[43] Jaiswal N Agrawal S Agrawal A. High fructose-induced metabolic changes enhance inflammation in human dendritic cells. Clin Exp Immunol (2019) 197(2):237–49. doi: 10.1111/cei.13299 PMC6642875 30919933

